# Inhibition of In Vitro Fertilizing Capacity of Cryopreserved Mouse Sperm by Factors Released by Damaged Sperm, and Stimulation by Glutathione

**DOI:** 10.1371/journal.pone.0009387

**Published:** 2010-02-24

**Authors:** Mary L. Bath

**Affiliations:** Division of Molecular Genetics of Cancer, Walter and Eliza Hall Institute of Medical Research, Parkville, Australia; Fred Hutchinson Cancer Research Center, United States of America

## Abstract

**Background:**

In vitro fertilization (IVF) of eggs by frozen and thawed C57BL/6J mouse sperm is inhibited by dead sperm and enhanced by preincubation of the sperm in calcium-free medium. In other species, the presence of sperm killed by freezing and thawing has been associated with the generation of hydrogen peroxide.

**Methodology/Principal Findings:**

The proportion of eggs fertilized by cryopreserved C57BL/6J mouse sperm was increased significantly by increasing the volume of fertilization medium in which sperm and eggs were coincubated. Enhanced fertilization occurred even though the concentration of potentially fertile sperm was decreased fivefold. This suggested that if a putative soluble factor was inhibiting fertilization, dilution of that factor, but not the sperm, should increase the fertilization rate. This was achieved by coincubation of the gametes in cell culture inserts (Transwells^®^) that during incubation were transferred progressively to wells containing fresh fertilization medium. Fertilization rates using inserts were high (66.6±2.4% versus 27.3%±2.8% in wells alone). On the assumption that the soluble factor could be H_2_O_2_, reduced glutathione was added to the fertilization medium. This enhanced fertilization rate significantly (76.6%±2.0% versus 21.2%±1.9%), while addition of oxidized glutathione did not (82.7%±6.5% with reduced glutathione; 44.5±8.8% with oxidized glutathione; 47.8%±12.1% with no glutathione). Positive effects of reduced glutathione on IVF were also seen with frozen 129S1, FVB, and C3H sperm, and sperm from two lines of genetically modified C57BL/6J mice.

**Conclusions/Significance:**

IVF in cell culture inserts and addition of glutathione to fertilization medium significantly increased the proportion of eggs fertilized by cryopreserved mouse sperm from four inbred strains, suggesting that reactive oxygen species generated during fertilization inhibit fertilization. The modified IVF techniques developed here enhance the feasibility and efficiency of using cryopreserved sperm from genetically modified lines of inbred mice.

## Introduction

The capacity of frozen and thawed mouse sperm to fertilize eggs in vitro appears to be inhibited by the presence of damaged sperm in the fertilization milieu [1]. Consequently, sperm suspensions from strains prone to sperm damage after cryopreservation, such as C57BL/6J (>80% damaged sperm) fertilize relatively few eggs (<20%), while those from strains producing few damaged sperm, such DBA/2 (<12% damaged sperm) fertilize a high proportion of eggs (>90%) [2]. Despite damage, a subpopulation of C57BL/6J sperm retains the potential to fertilize a high percentage of eggs. That potential is realized if sperm are incubated in calcium-free medium [1], [3], in medium containing methyl-beta-cyclodextrin (MBCD) [4], or in medium containing a mix of MBCD plus reducing agents [5], before transfer of selected motile sperm to the fertilization milieu.

In the current study, instead of selecting motile sperm, the effect of reducing the concentration of molecules released into the fertilization milieu during fertilization was investigated by incubating the sperm and eggs in cell culture inserts, without pre-incubation. Medium in the well below the inserts acted as a sink into which soluble factors could diffuse, to be diluted and removed from contact with sperm and eggs by subsequent transfer of inserts at intervals to wells containing fresh medium. This procedure resulted in high fertilization rates and suggested that a factor released into the fertilization milieu could be inhibiting fertilization.

Bovine sperm contain an aromatic amino oxidase that becomes active after sperm death [6], producing hydrogen peroxide, which reduces the lifespan of motile sperm, and which effect is eliminated by catalase, an antioxidant that converts hydrogen peroxide to water. Equine sperm damaged by 3 cycles of flash-freezing also generate increased amounts of H_2_O_2_ compared to fresh sperm [7]. This suggested that mouse sperm damaged by freezing and thawing might release hydrogen peroxide into the fertilization milieu, inhibiting fertilization.

To counteract any hydrogen peroxide produced, reduced glutathione (GSH) was added to the fertilization medium. Glutathione, a disulfide reductant with multiple functions in cells [8], [9] and multiple effects on sperm in vitro [10], was used because it previously had been included in an in vitro fertilization medium specifically for mice, although the reason was not discussed [11]. Based on a favorable outcome using C57BL/6J sperm, the investigation was extended to include 129S1/SvImJ, FVB/NJ, C3H/HeJ sperm, and sperm collected from 2 genetically modified lines with compromised in vivo fertility.

## Materials and Methods

### Animals

Mice were purchased from The Walter and Eliza Hall Institute's mouse breeding colony. They were maintained in accordance with the guidelines set out in the Australian Code of Practice for the Care and Use of Animals for Scientific Purposes [12], and were exposed to 14 h of light and 10 h of darkness each day. The experimental protocol was approved by the Animal Ethics Committee of The Walter and Eliza Hall Institute.

The strains used were C57Bl/6J, 129S1/SvImJ (129S1), FVB/NJ (FVB) and C3H/HeJ (C3H) and 2 strains of genetically engineered mice, haploinsufficient C57BL/6J *Bcl-x* [13] and heterozygous transgenic C57BL/6J VavP-*Myc*17 [14]. Both of the genetically engineered strains have reduced in vivo fertility caused primarily by low sperm concentration. The reduced fertility of the haploinsufficient *Bcl-x* mice results from loss of germ cells around embryonic day 13.5 and development of testicular hypoplasia after birth. The heterozygous transgenic VavP-*Myc*17 mice succumb to thymic T-cell lymphomas within 80 days of birth and produce few or no offspring.

Two-cell embryos were transferred into day 0.5 pseudopregnant (CBA X C57BL6/J) F1 female mice.

### Experimental Design

#### Experiment 1: Effect of dilution/removal of putative inhibitory molecules in fertilization medium

Sperm and eggs derived from C57BL/6J mice were incubated in cell culture inserts as described below to reduce the concentration of molecules released by the thawed sperm into the fertilization medium (FM). As it was not known if use of inserts would have the desired effect, especially if the sperm concentration was high, fertilization was done in two different sized wells (6.5 mm and 12.0 mm), with and without well inserts, using the same volume of sperm, and using sperm that had been extended in 4 different volumes of cryoprotectant (CPA). The sperm were divided into 4 treatment groups based on the volume of CPA used to extend the sperm (105 µL, 180 µL, 400 µL and 800 µL). Each group was comprised of sperm from 4 mice (4 extender treatment groups ×4 mice/treatment  = 16 mice). Samples (4) of sperm from each of the same mice were used for fertilization studies in the 6.5 mm and 12 mm inserts and wells. To confirm the observations made in determining optimal conditions, fertilization rates in 6.5 mm inserts and wells were compared in a second trial, using duplicate samples of sperm from 10 mice, extended in 180 µL of CPA.

#### Experiment 2: Effect of GSH on in vitro fertilization

Sperm were collected from C57BL/6J, 129S1, FVB and C3H mice and extended in 105 µL, 180 µL, 400 µL and 800 µL of CPA. The sperm from 4 mice of each strain were extended in each volume of CPA (4 mouse strains ×4 treatment groups/strain ×4 mice/treatment  = 64 mice).

For each strain the optimal concentration of GSH to include in the fertilization medium was determined using frozen sperm extended in 180 µL of CPA collected from a single mouse of each strain for each concentration of GSH, as outlined in Table 1.

**Table 1:** Dose-dependent effects of reduced glutathione on in vitro fertilization rate.^a^

GSH^b^ (mM)	Fertilization Rate (%)	Fertilization Rate (%)	Fertilization Rate (%)	Fertilization Rate (%)
	C57BL/6J^c^	129S1^c^	FVB/NJ^c^	C3H/HeJ^c^
2.00	84	nd^d^	nd	Nd
1.50	89	nd	nd	Nd
1.25	88	69	80	Nd
1.00	69	69	77	97
0.75	68	61	86	97
0.50	46	34	82	98
0.25	34	nd	48	98
0.00	07	14	50	73

a Sperm from one mouse of each strain were extended in 180 µL of cryoprotectant. Sperm (2 µL) and eggs (80−90) were co-incubated in 6.5 mm diameter tissue culture wells. The glutathione was added to the fertilization medium.

b Reduced glutathione.

c Mouse strain.

d Not done.

To evaluate the effect of GSH across strains under various conditions, fertilization was done in 6.5 mm wells (100 µL of medium) and 12.0 mm diameter wells (500 µL of medium) with 2 µL sperm and 80−90 eggs in medium supplemented with the optimized concentration of GSH and in medium lacking GSH. Sperm and eggs were from the same strain of mouse. The fertilization rates in FM + GSH and FM − GSH were compared. Since the C57BL/6J sperm samples were the same as those used in Experiment 1, the negative GSH controls used in that experiment (fertilization rates in 6.5 mm wells and 12.0 mm wells in FM lacking GSH) were used in this experiment.

To confirm the observations made in the experiment described above, fertilization rates using C57BL/6J sperm extended in 180 µL (n = 4), 300 µL (n = 4), and 400 µL (n = 4) in 6.5 inserts in FM + GSH and FM − GSH were determined, the results pooled (n = 12), and the difference between the means was evaluated.

All eggs were examined for morphological changes 6 h after incubation in FM − GSH or FM + GSH and after overnight incubation. Assessment of the changes was made by examining the zygotes/eggs at 20×−40× magnification.

#### Experiment 3: In vitro fertilization rates of cryopreserved sperm of two genetically engineered lines

The effect of GSH on fertilization rates using sperm from the reproductively compromised strains B6*Bcl-x* and B6VavP-*Myc* was evaluated in 6.5 mm wells + GSH and in 6.5 mm wells − GSH with 50–60 eggs and 2 µL of *Bcl-x* sperm suspension, or 4 µL of VavP-*Myc* sperm suspension. Despite the increased sperm volume, VavP-*Myc*17 sperm fertilized few eggs in 6.5 mm wells + GSH, so the fertilization rates with these sperm were determined using the alternative IVF procedure involving pre-incubation in calcium-free medium, described in Materials and Methods below.

#### Experiment 4: Parthenogenesis, fragmentation of eggs and birth of pups following embryo transfer

To ensure that embryogenesis was the result of fertilization, eggs were cultured in FM with and without killed sperm. The number of eggs that cleaved overnight was recorded.

The total number of eggs that fragmented during the fertilization and after overnight incubation was recorded.

The percentage of embryos produced under various conditions and transferred to pseudopregnant recipient mice that resulted in the birth of live pups was compared.

#### Experiment 5: Effect of equilibrating FM + GSH overnight at 37°C in 5.5% CO_2_/air

In the preceding experiments the FM was equilibrated in the CO_2_ incubator for a minimum of 1.5 h prior to the introduction of sperm and eggs. However, the observation that GSH is stable for at least 18 h in supplemented M199 at 37°C in an atmosphere of 5% CO_2_/air [15] led to a reappraisal of the need to equilibrate the medium immediately prior to IVF. Samples of sperm from the same 4 mice were subjected to each treatment. Sperm and eggs were co-incubated in FM + GSH, FM − GSH or in FM +1.25 mM GSSH (oxidized glutathione, G6654, Sigma). On the evening prior to the IVF procedure 625 µL of FM (with or without GSH or GSSH) was distributed into each well in 24-well plates; then a 6.5 mm insert was placed in each well, to which 100 µL of the appropriate FM was added. The plates were placed overnight in the CO_2_ incubator. The following morning 40−56 eggs denuded of cumulus cells and 3 µL of thawed C57BL/6J sperm that had been extended in 200 µL of CPA were placed in the insert and incubated for 6 h, after which the eggs were washed and incubated overnight. Fertilization rates were determined the following morning.

### Collection, Freezing, and Thawing of Sperm

Sperm were collected from virgin 4−8 month-old male wild-type C57BL/6J, 129S1, FVB and C3H mice. The two male *Bcl-x* mice were 3 month-old virgins. One suspension (S1) of VavP-Myc sperm was prepared from a mouse aged 51 days and the other suspension (S2) was prepared from all 4 epididymes of two mice aged 42 days.

Caudal epididymes were removed from sperm donors and both were placed in 105 µL, 180 µL, 400 µL or 800 µL of 23.7% (w/v) raffinose pentahydrate (R 7630, Sigma, St Louis, MO) solution in one well of a 4-well Multidish (#144444, Nunc, Roskilde, Denmark). Each epididymis was cut 5 or 6 times and the sperm were allowed to disperse for 1−2 min. The epididymal tissue was removed and the dish was gently shaken to encourage the sperm to distribute evenly. Then 14 µL volumes of the sperm suspension were transferred into 10, 0.25 mL freezing straws (IMV, L'Aigle, France) so that the trailing edge of the 7 mm sperm column was 1.0 cm from the open end of the straw after sealing. The end of the straw was sealed with straw sealing powder (Genetics Australia, Bacchus Marsh, Victoria, Australia). A Dewar flask (Model 5LD, Taylor Wharton, Theodore, AL) was filled with liquid nitrogen to within 8 cm of the rim, a Styrofoam lid was placed over the opening, and the sealed straws were placed vertically through individual holes in the lid so that the sperm column came to lie 18−25 mm above the liquid nitrogen. The sperm were left in the liquid nitrogen vapor for 5 min and then pushed through the holes into the liquid nitrogen where they floated until they were collected and stored in liquid nitrogen.

### Egg Collection

Eggs were collected from the mice following sequential intraperitoneal or subcutaneous injections of 7.5 IU equine chorionic gonadotrophin (eCG, Intervet, Victoria, Australia) and 7.5 IU human chorionic gonadotrophin (hCG, Intervet). The hormones were given 48 h apart to the C57BL/6J, C3H and FVB mice and 52−54 h apart to the 129S1 mice. On the day of the eCG injection the C57BL/6J mice were 26−29 days old; the 129S1, FVB and C3H mice were 65−80 days old. The hCG injection was given between 1545 and 1615 h on the day prior to IVF. At 14.5−15.5 h after the hCG injection the mice were killed humanely and their oviducts were removed. The cumulus masses were released from the ampullae into wash medium (see media details below) containing 0.03% hyaluronidase (H 3884, Sigma) to remove the cumulus cells. The eggs were washed free of hyaluronidase and then 80−90 eggs were transferred to each tissue culture insert or well containing FM (see details below) that had been pre-equilibrated in a humidified atmosphere of 5.5% CO_2_/air for at least 1.5 h.

### In Vitro Fertilization

#### Dishes and inserts

IVF in wells was carried out in 96-well and 48-well tissue culture plates containing 6.5 mm diameter wells (#167008, Nunc, Roskilde, Denmark) and 12.0 mm diameter wells (#15068, Nunc) respectively. The 6.5-mm inserts (#3413 Transwell, Corning Inc., Corning, NY) were placed over 24-well multiple well plates (Costar #3524, Corning) and 12.0-mm inserts (Transwell #3401) were placed over 12-well multiple well dishes (Costar #3512). The pore size of the polycarbonate membrane forming the base of the inserts was selected to facilitate passage of molecules released by the sperm, while excluding whole cells. Sperm and eggs were incubated overnight in 60 mm Petri dishes (#150288, Nunc).

#### Pipette Tips

Sperm and eggs were transferred into the wells and inserts with wide-bore 10-µL tips (#TF-400-LRS, Axygen Scientific, Union City, CA) and the eggs were washed out of the inserts and wells with wide-bore 200-µL tips (200G tips, # 2069G, Molecular BioProducts, San Diego, CA).

#### Media

The fertilization medium (Research Vitro Fert, K-RVFE-50), wash medium (Research Vitro Wash, K-RVWA-50) and mineral oil (K-SICO-200) were purchased from Cook Australia, Eight Mile Plains, Qld, Australia. On the afternoon prior to use, reduced glutathione (G6013, Sigma) was added to the FM (FM + GSH) which was stored overnight at 4°C. The concentration of GSH in the fertilization medium depended on the strain of mouse donating the sperm, as determined in Experiment 2. On the morning of the IVF, the fertilization media (FM – GSH and FM + GSH) were warmed to 37°C before distribution into dishes. The plates were then placed in an incubator with a humidified atmosphere of 5.5% CO_2_/air for 1.5 h or more before use.

In Experiment 5 the media were distributed into dishes on the evening prior to IVF and incubated overnight in the CO_2_ incubator. The media (FM +1.25 mM GSH and FM +1.25 mM GSSH) were prepared immediately before distribution into dishes. Calcium-free TYH medium [16], [17] was prepared weekly. One plate was used for each IVF. The fertilized eggs were incubated overnight in drops of fertilization medium containing 0.1 mM EDTA (E6511, Sigma) under oil.

#### Volume of sperm and number of eggs

Unless stated otherwise and regardless of the size of the insert or well, 80−90 eggs and 2 µL of sperm were used for each IVF replicate. Consequently, the concentration of sperm in the 12.0 mm inserts and wells was 5-fold lower than that in the 6.5 mm inserts and wells, and also varied depending on the volume of cryoprotectant (CPA) used to extend the sperm.

#### Fertilization in inserts

Tissue culture well inserts of two sizes, 6.5 mm and 12.0 mm diameter, were used for IVF. Four wells were used for each 6.5 mm insert and 3 wells for each 12.0 mm insert. The volume of FM placed in the well below the inserts was 625 µL for the 6.5 mm inserts and 1.5 mL for the 12.0 mm inserts. Fertilization medium (100 µL) was placed in the 6.5 mm inserts and 500 µL in the 12.0 mm inserts. If air bubbles were trapped in the well beneath, the insert was lifted and replaced or the plate was placed on its edge and gently tapped so that bubbles drifted to edge of the well and disappeared. The plates then were placed in the CO_2_ incubator to allow the medium to equilibrate.

Sperm were thawed by removing a straw from liquid nitrogen, holding it in room temperature air for 4 sec, then placing it in a water bath at 53°C for 6 sec. The 2 µL volume of sperm suspension was transferred to an insert or well containing eggs. Subsequently, inserts were transferred at 30 min intervals to wells containing fresh medium. Midway between transfers and 30 min after the last transfer, the plates containing the inserts were given a quick but brisk shake to lift cell debris off the porous base. The sperm and eggs were co-incubated for a total of 6 h before the eggs were removed and washed through 4 drops of wash medium and then incubated over night.

#### Fertilization in wells alone

Sperm and eggs were placed in 6.5 mm and 12.0 mm diameter wells containing 100 µL and 500 µL of FM or FM + GSH respectively. The plates were transferred to the incubator and the eggs and sperm were incubated for 6 h. Initially wells containing FM were removed from the incubator and treated as if they contained an insert. However, removal from the incubator was found to have no influence on the fertilization rate and the practice was abandoned.

Abbreviations used below for the various IVF protocols are: 6.5 mm insert − GSH; 6.5 mm well − GSH; 12.0 mm insert − GSH; 12.0 mm well − GSH; 6.5 mm well + GSH; 12.0 mm well + GSH.

#### In vitro fertilization protocol for VavP-Myc17 transgenic mice

Fertilization was done in a 6.5 mm insert. Instead of FM, calcium-free TYH medium was placed in one well (625 µL) and in the insert (100 µL). The plate was incubated overnight in the CO_2_ incubator. Next morning the plate was removed from the incubator and 625 µL of fertilization medium containing 0.75 mM GSH was added to 3 additional wells, and the plate was returned to the incubator for at least 30 min. After thawing, 4 µL of the sperm suspension was placed in the insert. The sperm were pre-incubated in the calcium-free TYH medium for 40−45 min and then 50−60 eggs were added to the insert. Immediately after adding the eggs the insert was transferred to a well containing GSH-supplemented fertilization medium, agitated twice, transferred to a second well of GSH-supplemented medium and agitated once more. Agitation ensured rapid transfer of the calcium-containing GSH-supplemented medium to the insert. The plate was returned to the CO_2_ incubator for another 30 min and then the insert was transferred to the third well containing GSH-supplemented medium. The plate was returned to the incubator. The gametes were co-incubated for a total of 6 hours. Eggs then were washed in wash medium, transferred to FM containing 0.1 mM EDTA and incubated overnight in the CO_2_ incubator.

#### IVF in 6.5 mm inserts in FM + GSH equilibrated overnight

The IVF was done in a 6.5 mm insert placed over one well in FM + GSH. The sperm and eggs were incubated for 6 h before the eggs were washed and incubated overnight.

### Incubation of Eggs with Killed Sperm

Parthenogenetic activation of eggs (embryogenesis in the absence of fertilization) was controlled for by incubating eggs with killed sperm for 6 h. The sperm were killed by thawing an aliquot of frozen sperm and then refreezing the sperm by plunging them directly into liquid nitrogen. The freezing and thawing was repeated once to ensure that all sperm were dead.

### Fertilization Rate

Fertilization rate was defined as the percentage of eggs inseminated that had cleaved 25 h after the insemination. Fragmented/degenerate embryos were considered as unfertilized eggs and were included in the calculation. Eggs that lost their zona pellucida during fertilization or overnight incubation (<1∶500) were not included in the calculation as it was possible that those eggs could be fertilized by sperm that had not passed through the zona pellucida [18].

### Embryo Transfer

Nine 2-cell embryos were transferred to the right oviduct of each pseudopregnant female on the day a vaginal plug was found (Day 1 of pseudopregnancy) and pups were born 19 days later.

### Statistics

Comparisons were made between the mean fertilization rates in inserts and wells of the same size and with sperm extended in the various volumes of CPA. Similarly, the mean fertilization rates following fertilization in FM − GSH and FM + GSH were compared. The comparisons were based on means derived from the fertilization rates determined once using sperm from 4 different mice (n = 4). The data were analyzed by ANOVA and the Tukey-Kramer test was used to determine the differences between means (GraphPad InStat, San Diego, CA) following arcsin transformation of the data. Differences were considered significant when P<0.05. The data are presented as mean ± SEM. When direct comparisons were made between fertilization rates in inserts and wells (n = 10) or in FM + GSH and FM (n = 12) the significance of the difference between the means was determined by the Student's paired sample t-test after arcsin transformation of the data. Differences between fertilization rates in 6.5 mm and 12.0 mm wells in FM − GSH for sperm extended in the same volume of CPA were determined using the Student's t-test.

## Results

### Experiment 1: Fertilization in Inserts and Wells Using C57BL/6J Sperm

**Figure 1:**
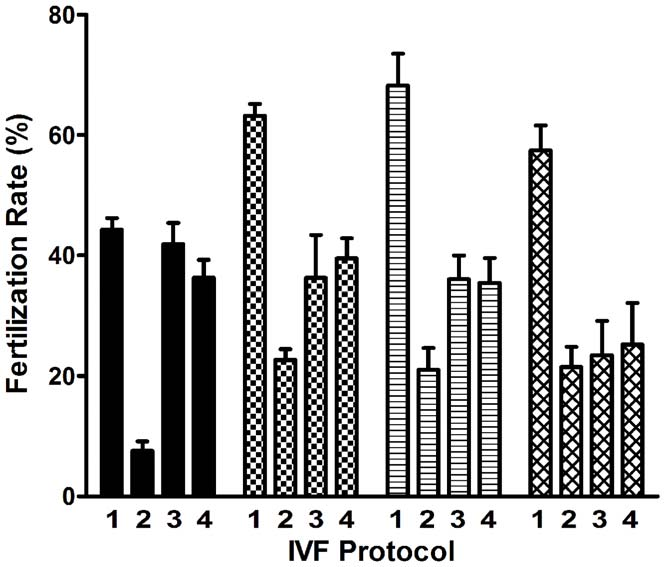
In vitro fertilization rates with cryopreserved C57BL/6J sperm in cell culture inserts and wells. The bar patterns, in groups of 4, indicate the volume of cryoprotectant used to extend the sperm prior to freezing. From L to R: 105 µL, 180 µL, 400 µL and 800 µL. Numbers within each group of 4 bars indicate IVF Protocol: Protocol 1, 6.5 mm insert; Protocol 2, 6.5 mm well; Protocol 3, 12 mm insert; Protocol 4, 12 mm well. Data are presented as mean ± SEM, n = 4. Significance of differences is described in text.

Fertilization rates in the 6.5 mm inserts were consistently higher than those in the 6.5 mm wells in all groups (P<0.001; Fig. 1). There was no significant difference in the fertilization rates in the 12.0 mm inserts and 12.0 mm wells within each group. Apart from the sperm extended in 800 µL of CPA, fertilization rates within each group were significantly higher in the 12.0 mm wells than in the 6.5 mm wells (36.3±3.0% vs. 7.6%±1.7, P<0.001; 39.5%±3.4% vs. 22.7%±1.8%, P<0.004 and 35.4%±4.1% vs. 21.0%±3.6%, P<0.036 for sperm extended in 105 µL, 180 µL and 400 µL of CPA respectively). The fertilization rate using sperm from 10 mice extended in 180 µL CPA was significantly higher in 6.5 mm inserts vs. 6.5 mm wells (66.6±2.4% vs. 27.3%±2.8% respectively; P<0.0001).

### Experiment 2: The Effect of GSH on In Vitro Fertilization

#### The concentration of GSH

Table 1 shows the concentration-dependent effects of GSH in the FM on the fertilization of eggs by frozen and thawed C57BL/6J, 129S1, FVB and C3H mouse sperm. Based on fertilization rates and the percentage of pups born after embryo transfer, the concentration of GSH determined to be optimal was 1.25 mM for C57BL/6J, 129S1 and FVB sperm and 0.5 mM for C3H sperm.

#### Fertilization rates using C57BL/6JB sperm

**Figure 2:**
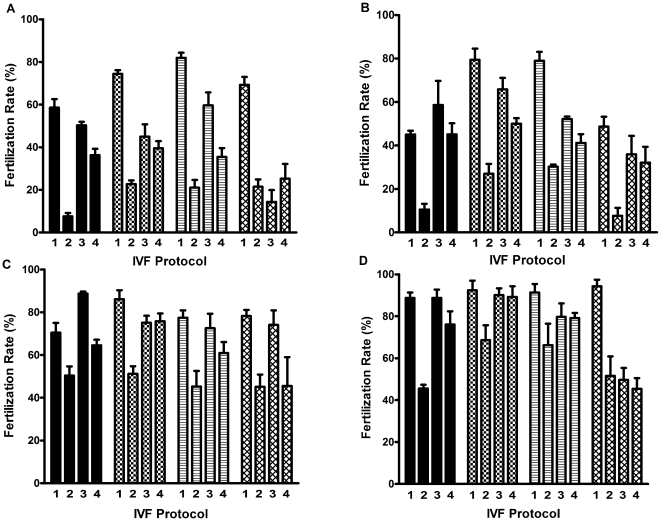
In vitro fertilization rates in medium containing reduced glutathione (GSH) and in medium lacking GSH. (A) C57BL/6J, (B) 129S1/svImJ, (C) FVB/NJ and (D) C3H/HeJ sperm, in fertilization medium containing reduced glutathione (GSH) and in medium lacking GSH. The bar patterns, in groups of 4, indicate the volume of cryoprotectant used to extend the sperm. From L to R: 105 µL, 180 µL, 400 uL and 800 µL. Numbers within each group of 4 bars indicate IVF Protocol: Protocol 1, 6.5 mm well + GSH; Protocol 2, 6.5 mm well − GSH; Protocol 3, 12 mm well + GSH; Protocol 4, 12 mm well − GSH. The fertilization medium was supplemented with 1.25 mM GSH for the C57BL/6J, 129S1, and FVB sperm and with 0.5 mM GSH for the C3H sperm. Data are presented as mean ± SEM, n = 4 and significance of differences is described in text.

For each extender volume, supplementation of the fertilization medium with 1.25 mM GSH increased the fertilization rate in 6.5 mm wells (P<0.001; Fig. 2A). In the 12.0 mm wells the increase was seen only for sperm extended in 400 µL of CPA (P<0.05).

The mean fertilization rate using sperm extended in 180 µL, 300 µL and 400 µL of CPA (n = 12) in 6.5 mm wells in medium containing GSH was significantly higher than in medium lacking GSH (76.6%±2.0% vs. 21.2%±1.9%, P<0.0001).

#### Fertilization rates using 129S1 sperm

Fertilization rates using 129S1 sperm (Fig. 2B) resembled those with C57BL/6J sperm. In 6.5 mm wells + GSH fertilization was consistently better than in 6.5 mm wells − GSH (P<0.01 to P<0.001). Addition of GSH did not increase the fertilization rate in the 12.0 mm wells. Fertilization rates in FM − GSH (IVF Protocols 2 and 4, Fig. 2B) were consistently higher in the 12.0 mm wells than in the 6.5 mm wells.

#### Fertilization rates using FVB sperm

Overall, fertilization rates using FVB sperm (Fig. 2C) were high compared to C57BL/6J and 129S1 sperm. However, the trends were similar; including a significant increase in the fertilization rate in 6.5 mm wells + GSH compared to those in 6.5 mm wells – GSH. Apart from the sperm extended in 105 µL of CPA, GSH had no significant effect on fertilization rates in 12.0 mm wells. Apart from sperm extended in 800 µL of CPA, fertilization rates within groups were significantly higher in 12.0 mm wells − GSH than in 6.5 mm wells − GSH.

#### Fertilization rates using C3H sperm

Overall, fertilization rates using C3H sperm were high (Fig. 2D). GSH increased the fertilization rate in the 6.5 mm inserts significantly but had no effect in the 12.0 mm inserts. Fertilization rates in medium with and without GSH were especially high (>70%) in the 12.0 mm wells using sperm prepared in 105−400 µL of CPA. The difference in fertilization rates in 12.0 mm and 6.5 mm wells in FM − GSH within groups was significant only for sperm extended in 105 µL of CPA.

#### Summary of fertilization rates in GSH

In general, fertilization rates achieved by FVB and C3H sperm were higher than those achieved by C57BL/6J and 129S1 sperm. Fertilization rates were highest in 6.5 mm wells in FM + GSH. In contrast, in FM – GSH, fertilization rates were higher in 12.0 mm wells than 6.5 mm wells despite the 5-fold lower sperm concentration in the 12.0 mm wells. Overall, fertilization rates achieved by sperm extended in 105 µL and 800 µL of CPA were lower than those extended in 180 µL and 400 µL of CPA.

#### Effect of GSH on the zona pellucida

**Figure 3:**
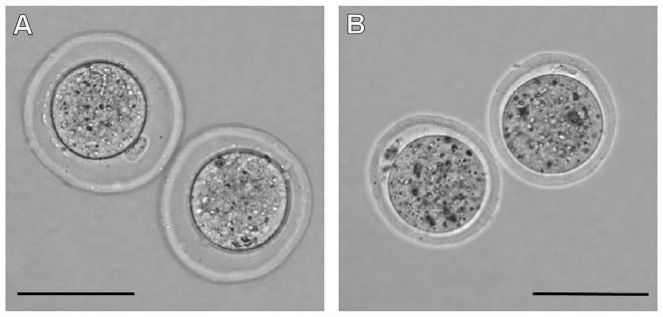
Effect of reduced glutathione (GSH) on mouse eggs. (A) C57BL/6J eggs incubated for 6 h in fertilization medium containing 1.25 mM GSH. The zona pellucida has expanded resulting in an increase in width of the perivitelline space, compared with eggs incubated medium lacking GSH (B). Each scale bar  = 100 µm.

A slight increase in the size of the perivitelline space caused by expansion of the zona pellucida was observed following IVF in fertilization medium containing GSH (Fig. 3). This appeared to be accompanied by thinning of the zona, although the latter was quite difficult to discern. The effect was variable and often more noticeable in eggs incubated in medium in the absence of sperm. The change in the zona was less obvious when the fertilization medium was equilibrated in the CO_2_ incubator overnight.

### Experiment 3: Fertilization Rates Using Bcl-X and Vavp-Myc17 Sperm

#### Bcl-x

Using IVF protocol 6.5 mm well − GSH the fertilization rates were 16% and 17% with the two sperm suspensions. The fertilization rates in medium containing 1.25 mM GSH were 60% for both suspensions in one experiment and 60% and 64% in a second experiment.

#### *VavP-Myc*17

In 6.5 mm wells − GSH the fertilization rates were less than 10% in two separate experiments for both suspensions. With GSH in the medium the fertilization rates were 18% and 31% with S1, and 29% and 43% with S2, in separate experiments. When the sperm were pre-incubated in 6.5 mm inserts in calcium-free medium and then transferred with the eggs to medium containing 0.75 mM GSH the fertilization rates on 3 separate occasions were 59%, 70% and 78% for S1, and 62%, 70% and 88% for S2.

### Experiment 4: Parthenogenetic Activation of Eggs

Eggs were incubated alone or with killed sperm in FM + GSH and in FM − GSH to check that embryogenesis was initiated by fertilization and not by an environmental factor. The addition of killed sperm made no difference and the results were pooled for subsequent analysis.

#### C57BL/6J

Of 439 eggs tested none developed into 2-cell embryos. One egg fragmented/degenerated overnight.

#### 129S1

Of 232 eggs, none developed into 2-cell embryos. Two eggs fragmented overnight.

#### FVB

Of 479 eggs incubated 3 (0.6%) cleaved and 47% fragmented overnight.

#### C3H

Of 118 eggs examined none cleaved and none fragmented overnight.

### Experiment 4: Fragmented Eggs

In calculating the fertilization rate fragmented eggs were considered to be unfertilized. Up to 80% of unfertilized FVB eggs in any one IVF trial fragmented. In contrast, less than 5% of unfertilized B6, 129S1 and C3H eggs fragmented.

The presence of GSH in the fertilization medium had no influence on the percentage of eggs that fragmented.

### Experiment 4: Birth of Pups

The female offspring produced were used as egg donors in subsequent experiments. There was no difference in the percentage of pups retrieved following transplantation of eggs fertilized in inserts and in wells, so the results were pooled for subsequent analysis.

#### C57BL/6J

There was no difference in the percentage of pups born (130/268 vs. 64/130, both 49%) after embryo transfer of eggs fertilized in FM +1.25 mM GSH and FM − GSH.

#### 129S1

Of 880 embryos transferred from eggs fertilized in FM +1.25 mM GSH 62% developed into pups, while 65% of 535 embryos from eggs fertilized in FM − GSH developed into pups.

#### FVB

Fertilization in FM +1.25 mM GSH increased the percentage of FVB/NJ embryos that developed into pups in comparison with fertilization in FM − GSH (52% of 684 vs. 35% of 517 eggs transferred).

#### C3H

Of 648 eggs transferred following fertilization in FM +0.5 mM GSH, 221 (34%) developed into live young. Following IVF in FM − GSH 192 pups (36%) were born from 531 embryos transferred.

### Experiment 5: Fertilization Rates in Medium Equilibrated Overnight Using C57BL/6J Sperm

Fertilization rates in 6.5 mm inserts (without transfer to fresh wells) in medium containing 1.25 m mM GSH, no GSH, and 1.25 mM oxidized GSH for sperm from 4 mice were 82.7%±6.5%, 47.8%±12.1% and 44.5±8.8% respectively. The fertilization rate in medium containing GSH was significantly higher than in medium containing GSSH or no GSH (P<0.05).

## Discussion

It is clear from experiments using inserts that soluble factors released into the fertilization milieu by frozen/thawed C567Bl/6J sperm reduced fertilization by potentially competent sperm present in the fertilization medium. Fertilization rates in wells also were increased by diluting the sperm, up to a point. The same trend was observed for the sperm of 129S1, FVB and C3H mice, suggesting that freezing and thawing mouse sperm of many strains generally produces similar inhibitors of fertilization.

Increased fertilization in response to GSH supports the hypothesis that the inhibitors are reactive oxygen species (ROS) released by dead/moribund sperm or evolved in their presence. It is possible that the ROS perturb the function of thiol-sensitive proteins involved in sperm capacitation [4]. These proteins are probably situated on the sperm surface or in the fertilization medium and may be those required for removal of cholesterol from sperm [4]. It is conceivable that the function of these proteins is preserved or restored by GSH acting as a thiol reductant [10], [19], [20]. However, this is speculative, and the mechanism by which GSH and other antioxidants [5], [21], [22] restore the fertility of mouse sperm after cryopreservation is not known.

The addition of monothiolglycerol (MTG) to a commonly used cryoprotectant (18% raffinose, 3% skim milk), has been shown to increase the percent of eggs fertilized by C57BL/6J sperm [21]. Increased fertilization occurred only after the sperm had been pre-incubated in fertilization medium before the addition of eggs. Inclusion of 1.0 mM GSH in the cryoprotectant used in the current study did not increase fertilization rates possibly because the sperm were not pre-incubated. However, addition of MTG to FM, in concentrations ranging from 0.5−2.5 mM, increased fertilization rates to percentages comparable to those observed in FM + GSH. The experiments using MTG were done once using C57BL/6J sperm and eggs (data not shown).

Pre-incubation of VavP-*Myc*17 sperm in “calcium-free” medium prior to the introduction of eggs and transfer to medium containing GSH increased sperm fertility by an unknown mechanism. It is possible that this treatment prevented acrosomal loss, as has been seen in cryopreserved C57BL/6J sperm [3], or increased the fertile lifetime of the sperm by preventing promiscuous uptake of calcium [23].

Ostermeier et al [21] and Taguma et al [5] reported that fertilization rates varied with mouse strain and that these strain effects remained despite improved IVF protocols. A similar trend was noted in the current study but was eliminated by extending the sperm in 180−400 µL of CPA and co-incubating the gametes in 100 µL of FM + GSH. The fertilization rates achieved by the 129S1 sperm used in the current study were significantly higher than those reported by Ostermeier et al for this strain [21]. The difference may relate to the age of the egg donors used; 70-days in the current study, and <28 days in the former study [21].

Following fertilization in medium supplemented with GSH, eggs with an enlarged perivitelline space were observed. This was most likely caused by reduction of the disulfide bonds connecting individual glycoprotein filaments (GP1 filaments) involved in maintaining the three dimensional structure and rigidity of the zona [24]. The zonae of eggs exposed to GSH were less rigid than those of unexposed eggs but they remained intact and therefore could only be penetrated by capacitated sperm [25]. However, the altered zonae may have enhanced fertilization efficiency by facilitating transfer of the sperm to the perivitelline space [26].

In this study several procedures were shown to improve the capacity of cryopreserved mouse sperm to fertilize eggs in vitro, and it is unlikely that a single protocol will consistently result in high fertilization rates, particularly if the sperm are derived from genetically modified lines [21], [27]. However, for a one-protocol-fit-all approach the following is suggested. Extend the sperm from a single mouse in 200 µl of 23.7% (w/v) raffinose pentahydrate and freeze by placing the suspension 18−25 mm above liquid nitrogen in an enclosed container. If the straws are placed on a horizontal support ensure that the sperm suspension is placed over the edge of the support. On the evening prior to the IVF add GSH to the fertilization medium (final concentration 0.25 mM to 1.25 mM depending on the genetic background of the sperm). After warming, transfer 625 µl of FM + GSH into a well of a 24-well plate, place a 6.5 mm insert in the well and add 100 µl of the same medium to the insert. Leave the plate on a bench for a few minutes and then remove air bubbles trapped under the insert. Transfer the plate to the incubator. Next morning add 3 µl of sperm, incubate 40−45 min, give the plate a brisk shake then add 50−80 cumulus-free eggs and incubate for 6 h. Wash zygotes/unfertilized eggs to remove sperm. Incubate the eggs overnight and then transfer the 2-cell embryos to pseudopregnant recipient mice. Pre-incubation of the sperm is not necessary. However, it minimizes the time the sperm are away from the incubator, allows time for the FM to re-equilibrate before adding eggs, and does not reduce fertilization rates. It is important to use an incubator with a rapid recovery of CO_2_ (0 to 5.5% within 12 min) and one that sustains minimal loss of CO_2_ after the door is opened. Pre-incubation of the sperm is not recommended in FM − GSH unless the medium is calcium-free. Pre-incubation in calcium-free medium may increase fertilization rates if the above procedure is not successful.

In summary, several procedures that increase the in vitro fertility of cryopreserved mouse sperm were described. The procedures were shown to be applicable to a variety of inbred strains and to 2 lines of genetically modified mice known to have reduced fertility. Recovery of pups following transfer of 2-cell embryos to pseudopregnant recipients was not compromised by the procedures. The most significant finding was that supplementation of the fertilization medium with reduced glutathione significantly increased the capacity of the sperm to fertilize eggs in vitro.
